# *QuickStats:* Percentage* of Children and Adolescents Aged 1–17 Years Who Received an Influenza Vaccine Within the Past 12 Months,^†^ by Health Insurance Coverage^§^ and Age Group — National Health Interview Survey, United States, 2019–2020^¶^

**DOI:** 10.15585/mmwr.mm705152a4

**Published:** 2021-12-31

**Figure:**
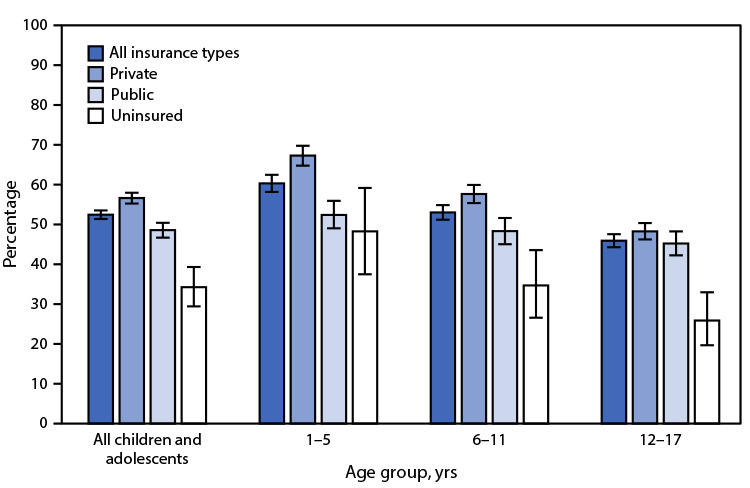
Throughout calendar years 2019–2020, 52.4% of children and adolescents aged 1–17 years received an influenza vaccine within the previous 12 months. The percentage was highest in children and adolescents with private insurance (56.6%), followed by those with public insurance (48.6%), and lowest in the uninsured (34.2%). This pattern was seen in each age group. The percentage of children and adolescents who received an influenza vaccine decreased with increasing age from 60.3% in children aged 1–5 years, to 53.0% in those aged 6–11 years, to 45.9% in adolescents aged 12–17 years. The decrease with age group was seen for each insurance type. Privately insured children aged 1–5 years had the highest rate of influenza vaccination, and uninsured adolescents aged 12–17 years had the lowest rate.

**Source:** National Center for Health Statistics, National Health Interview Survey, 2019**–**2020. https://www.cdc.gov/nchs/nhis.htm

For more information on this topic, CDC recommends the following link: https://www.cdc.gov/flu/prevent/vaccinations.htm

